# Touch-actuated microneedle array patch for closed-loop transdermal drug delivery

**DOI:** 10.1080/10717544.2018.1507060

**Published:** 2018-09-05

**Authors:** Jingbo Yang, Zhipeng Chen, Rui Ye, Jiyu Li, Yinyan Lin, Jie Gao, Lei Ren, Bin Liu, Lelun Jiang

**Affiliations:** a Guangdong Provincial Key Laboratory of Sensor Technology and Biomedical Instrument, School of Biomedical Engineering, Sun Yat-Sen University, Guangzhou, PR China;; b Department of Mechanical and Biomedical Engineering, City University of Hong Kong, Hong Kong, PR China

**Keywords:** Transdermal drug delivery, microneedle array, traditional transdermal patch, insertion, diabetes, insulin, closed-loop control

## Abstract

To date, only approximately 20 drugs synthesized with small molecules have been approved by the FDA for use in traditional transdermal patches (TTP) owing to the extremely low permeation rate of the skin barrier for macromolecular drugs. A novel touch-actuated microneedle array patch (TMAP) was developed for transdermal delivery of liquid macromolecular drugs. TMAP is a combination of a typical TTP and a solid microneedle array (MA). High doses of liquid drug formulations, especially heat-sensitive compounds can be easily filled and stored in the drug reservoir of TMAPs. TMAP can easily penetrate the skin and automatically retract from it to create microchannels through the stratum corneum (SC) layer using touch-actuated ‘press and release’ actions for passive permeation of liquid drugs. Comparison of subcutaneous injection, TTP, solid MA, and dissolvable MA, indicated that insulin-loaded TMAP exhibited the best hypoglycemic effect on type 1 diabetic rats. A ‘closed-loop’ permeation control was also provided for on-demand insulin delivery based on feedback of blood glucose levels (BGLs). Twenty IU-insulin-loaded TMAP maintained the type 1 diabetic rats in a normoglycemic state for approximately 11.63 h, the longest therapeutic duration among all previously reported results on microneedle-based transdermal patches. TMAP possesses excellent transdermal drug delivery capabilities.

## 1. Introduction

The transdermal drug delivery system (TDDS) describes the system that releases a drug from a specially designed device that diffuses through various layers of skin and into the systemic circulation to exert its therapeutic effects (Prausnitz and Langer, 2008; Han and Das, 2015; Ma and Wu, 2017). The TDDS has many advantages for patients, including its noninvasive and convenient nature, the avoidance of first-pass metabolism and the prevention of gastrointestinal degradation (Herwadkar and Banga, 2012; Larrañeta et al., 2016; Yu et al., 2017c). Traditional transdermal patches (TTP) have been applied in the medical treatment for patients owing to the simplicity of self-administration, painless response, improved patient compliance, and ease of disposal (Alexander et al., 2012; Gratieri et al., 2013). TTP usually contains a drug reservoir, which can store a large dose of drugs, thereby guaranteeing continuous drug release and long-term maintenance of relatively constant plasma concentration with the same patch (Larrañeta et al., 2016). However, to this-date, only approximately 20 transdermal drugs have been approved by the FDA for TTP (Kim et al., 2018). Moreover, the molecular weights of the drugs used thus far are below 400 Da (Ma and Wu, 2017). This is attributed to the skin’s excellent barrier function (Liu et al., 2016; Ono et al., 2017; Zhu et al., 2017). The stratum corneum (SC) of the skin is a protective barrier, which prevents most drugs or therapeutic agents to enter deeper skin layers, particularly high molecular weight molecules (Tuan-Mahmood et al., 2013; Lee et al., 2017). Only small molecular drugs (<400 Da) can generally cross the skin at therapeutic rates while the dose of these drugs that permeate through the skin is relatively low (Li et al., 2017b).

Numerous conventional transdermal techniques have been reported to alter the skin barrier to enhance the permeability of macromolecular drugs across the skin. Subcutaneous injection has been used to deliver the macromolecular drugs across the skin. Despite its extensive use, this technique has disadvantages, including accidental needle-sticks, risks of infection, bleeding, pain, and needle phobia (Indermun et al., 2014; Park et al., 2016; Raphael et al., 2016; Vinayakumar et al., 2016). Other techniques, including chemical enhancers (Pham et al., 2016; Liu et al., 2017), thermal ablation (Sawyer et al., 2009; Lee et al., 2011), electroporation (Wong, 2014), iontophoresis (Charoenputtakun et al., 2015), sonophoresis (Park et al., 2014; Zorec et al., 2015), have been developed to increase the permeability of the SC layer. However, these techniques suffer from the risk of skin irritation, safety issues, usage of sophisticated devices, or economic terms (Wong, 2014; Larrañeta et al., 2016; Wang et al., 2017). During the last few decades, the microneedle array (MA) has emerged as one of the most promising TTDS in the pharmaceutical field and has received considerable attention (Park et al., 2010; Shin, 2017). MA has demonstrated enhanced therapeutic efficacy by delivering macromolecular compounds across the skin barrier (Arora et al., 2008; Ma and Wu, 2017).

MA is a minimally invasive tool that penetrates the SC layer to create microchannels and increase skin permeability, thereby permitting entry of macromolecular drugs into the systemic circulation. MA has many advantages, such as pain-free delivery, minimal skin trauma, lack of bleeding or introduction of pathogens, and ease of disposal (Caffarel-Salvador and Donnelly, 2015; Larrañeta et al., 2016). Currently, approximately five basic types of MA have been developed for transdermal drug delivery, named porous MA, solid MA, coated MA, dissolvable/degradable MA (DMA), and hollow MA (van der Maaden et al., 2012; Tuan-Mahmood et al., 2013; van der Maaden et al., 2015; Larrañeta et al., 2016). These MAs have key features, yet they also have some limitations, which prevent their market spreading. The porous MA suffers from insufficient mechanical strength, resulting in a risk of breakage in the skin (Li et al., 2017a). The solid MA has a relatively high mechanical strength but is limited by its ‘poke with patch’ two-step delivery process, which leads to practical issues for patients (Wang et al., 2016). The major problem of coated MA is the low-payload capability (Larrañeta et al., 2016). The DMA is restricted by the dose of loaded drugs in the finite microneedles and the capacity for delivery of liquid drugs (Indermun et al., 2014). The hollow MA can continually deliver liquid drugs, but is limited by the complex fabrication process, potential microneedle clogging, and requirement of a syringe to inject liquid formulations (Kim et al., 2012; Yan et al., 2013). Above all, the development of an MA transdermal patch with increased mechanical strength, that is easily self-administered, has low cost, and is capable of delivering large doses of liquid macromolecular drugs, is still a challenge.

In this study, we present a novel MA transdermal patch named touch-actuated microneedle array patch (TMAP) for on-demand dosage delivery of liquid macromolecular drugs. TMAP is a typical TTP integrated with a solid MA. It inherits both key features, in that solid MA creates microchannels through the SC layer by touch-actuated ‘press and release’ actions for passive-diffusion driven delivery of liquid drugs from the drug reservoir of TTP. A high dose of liquid compounds, including heat-sensitive macromolecular drugs (such as insulin, proteins, vaccines, DNA, antibodies, and so on), can be easily loaded in the drug reservoir for transdermal delivery. The drug administration of TMAP can achieve on-demand dosage control via the reopening and self-closure of microchannels in the skin. TMAP is an attractive candidate for transdermal drug delivery.

## 2. Experimental

### 2.1. Ethics statement

All animal procedures conducted in this work were reviewed, approved, and supervised by the Institutional Animal Care and Use Committee (IACUC) at the Sun Yat-sen University (Approval Number: IACUC–DD–16–0904).

### 2.2. Materials and animals

Medical tape with an antiseepage gasket and medical sponge were purchased from Zhengkang Healthcare Co., Ltd, China. The antiseepage gasket is directly bonded on the medical tape. The detailed parameters of the medical tape, gasket, and sponge, are listed in Table S1. Rhodamine B was purchased from Aladdin, China. Streptozotocin (STZ, Sigma, USA) and insulin (bovine pancreas, CAS: 11070–73–8, Shanghai, China) were purchased for *in vivo* diabetic rat treatments.

Fresh rabbit skin was prepared for mechanical and transdermal drug diffusion tests. A New Zealand rabbit (male, age = 2–3 months old, and weight = 3.0 kg) was bought from the Xinhua Experimental Animal Farm (Huadu District, Guangzhou, China). The rabbit was humanely euthanized by intravenous injection of pentobarbital. The hair was shaved off and the subcutaneous fat was removed. The skin was cut into squares with sizes of 50 mm × 50 mm and thicknesses of 1.4 ± 0.1 mm. Sprague–Dawley (SD) rats (female, 200 ± 30 g) were supplied by the Experimental Animal Center of Sun Yat-sen University, China.

### 2.3. Fabrication of TMAP

**Figure 1.**
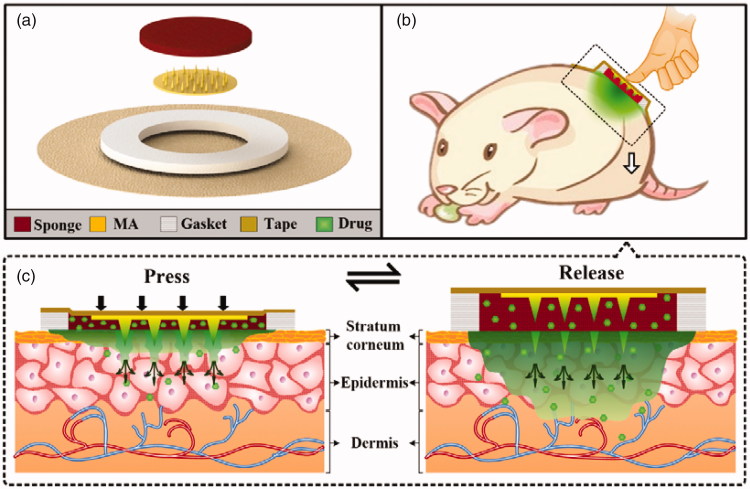
(a) Expanded view of TMAP components. (b–c) Schematic representation of the drug administration strategy of TMAP.

The exploded diagram of TMAP is presented in Figure 1(a). It consisted of a solid MA, a medical tape, an anti-seepage gasket, and a medical sponge. The polymethyl methacrylate (PMMA) solid MA was fabricated using a micromolding technique (Li et al., 2017b). The detailed fabrication process of the solid MA is presented in Figure S1 of Supporting Information. MA was directly bonded on the medical tape at the center of the anti-seepage gasket. A circular sponge was assembled in the inner circle of the gasket and bonded on the medical tape. The sponge acted as the liquid drug reservoir. A certain amount of liquid drug was directly added in a dropwise manner and was absorbed by the sponge. TMAP was finally assembled. TMAP was observed with a scanning electron microscope (SEM, Quanta 400 F, Oxford, Holland) and a digital camera (Canon, Guangzhou, China).

### 2.4. Mechanical tests

A custom-made mechanical loading setup was developed for the mechanical test of TMAP, as shown in Figure S2. A ‘press and release’ test was performed: (1) the sponge of TMAP was filled with 150 μL rhodamine B solution. (2) Under a relative humidity of 80%, a fresh rabbit skin was fixed on a polystyrene foam, which was used to mimic the soft tissue under the skin (Chen et al., 2016). (3) TMAP was slightly taped on the skin. (4) Press stage: the compression plate was driven towards TMAP by a linear motor with a loading displacement of 3 mm and a velocity of 0.1 mm s^−1^. (5) Hold stage: the linear motor was stopped at the loading displacement of 3 mm for 10 s. (6) Release stage: the compression plate was moved upwards at a velocity of 0.1 mm s^−1^ with an unloading displacement of 3 mm. (7) The force and displacement were recorded synchronously. (8) TMAP was peeled off from the punctured skin within a period of 10 min. The punctured skin was prepared for histology by freezing it in O.C.T Compound (SAKURA, Tissue-Tek ®American, Torrance, CA, USA) at −25 °C, and subsequently slicing it into sections with a 12 μm thickness with a cryostat microtome (Leica, CM1850UV, Nussloch, Germany). (9) The skin slices were observed with an inverted fluorescence microscope (Eclipse Ti-E, Nikon, Tokyo, Japan). The minimum force required for the microneedle penetration into the skin was defined as the penetration force (Cho et al., 2012; Ling et al., 2017).

**Figure 2.**
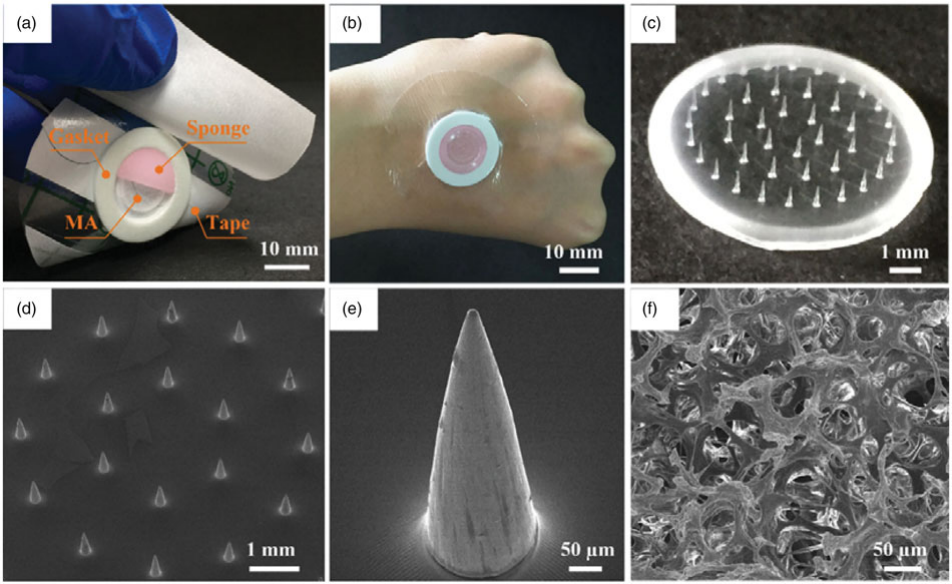
(a) Photo of TMAP and its components, (b) image of TMAP taped on the back of a human hand, (c) photo of solid MA, (d) SEM image of solid MA, (e) SEM image of one of the microneedles, and (f) SEM image of medical sponge.

The mechanical stability of TMAP was assessed by multiple insertions. The ‘press and release’ test of TMAP was repeated for five times at each specific spot of the rabbit skin. The punctured pores in the skin were observed with a microscope. The mechanical stability could be evaluated by the relationship between the percentage of the micropores created by TMAP and its microneedle number (Lee et al., 2008). This TMAP was peeled off and taped at another location of the rabbit skin and poked five more times. The punctured skin was observed again. This process was repeated for 12 cycles.

### 2.5. Stability of insulin in TMAP

To determine the storage stability of insulin solution encapsulated in TMAP, 150 μL insulin-loaded TMAP samples were stored at room temperature (approximately 25 °C) and at 4 °C for 90 d, respectively. Their bioactive insulin concentrations were measured using an insulin ELISA kit (Jianglai industrial Ltd., Shanghai, China), according to standard protocols (Yu et al., 2017a; Yu et al., 2017c).

### 2.6. *In vitro* transdermal drug delivery test

*In vitro* transdermal drug diffusion performance of TMAP was investigated in comparison with the solid MA and TTP. A TMAP without the use of a solid MA assembly was employed as a TTP. (1) Rhodamine B solution (150 μL) was used as a model drug and filled the sponge pores of TMAP and TTP. (2) TMAP loaded with rhodamine B was taped on fresh rabbit skin. The ‘press and release’ actions were repeated for five times. They managed to maintain the diffusion of rhodamine B into the skin for 10 min. Three cycles were performed within a total duration of 30 min. (3) Solid MA was poked in rabbit skin under a maximum compression force of 5 N. TTP loaded with rhodamine B was patched on the punctured skin for 30 min following removal of solid MA. This is a typical delivery approach of solid MA commonly referred to as ‘poke with patch.’ (4) TTP loaded with rhodamine B was patched on the intact rabbit skin for 30 min. (5) These skin samples diffused with rhodamine B were embedded in O.C.T Compound (SAKURA, Tissue-Tek ^®^American, Torrance, CA, USA), frozen at −25 °C, and cut into slices with a thickness of 10 μm every 35 μm-depth using a cryostat microtome (Leica, CM1850UV, Nussloch, Germany). (6) The skin slices were observed with an inverted fluorescence microscope (Eclipse Ti-E, Nikon, Tokyo, Japan). The fluorescent area of the slices where rhodamine B diffused was measured with ImageJ (National Institutes of Health, Bethesda, MD, USA).

### 2.7. Transepidermal water loss (TEWL)

TEWL measurement has been used to assess the disruption level of skin barrier function (Zhou et al., 2010; Donnelly et al., 2012; Ling and Chen, 2013). SD rats were used in this experiment after their hair on the back region was shaved, as shown in Figure S3(a). The rats were then fed for another day. The insulin-loaded TMAP was then patched on the rat skin and the ‘press and release’ was performed five times. The TEWL of the punctured skin following the removal of TMAP was measured with a vapometer (Delfin Technologies, Kuopio, Finland ) for a period up to 1 h by comparing it with the TEWL tested on the intact skin of the same rat, according to previous reports (Donnelly et al., 2012).

**Figure 3.**
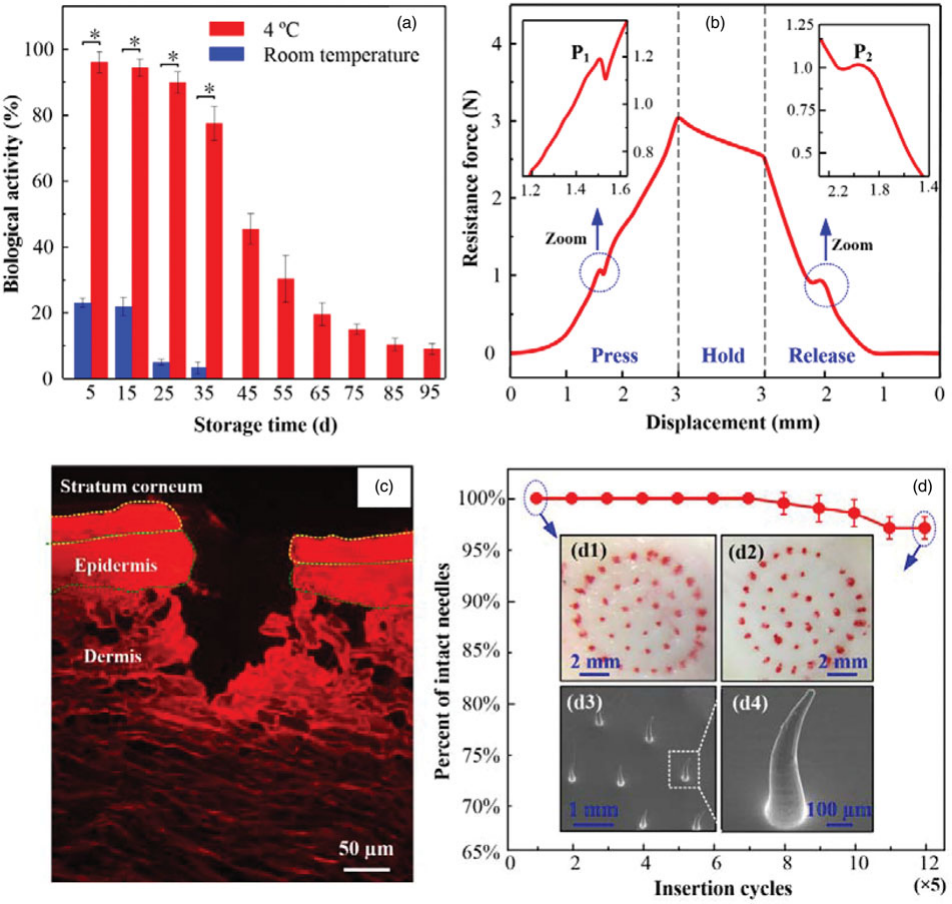
(a) Biological activity of insulin in TMAP stored at room temperature and at 4 °C. (b) The relationship between the resistance force and the loading displacement during the ‘press and release’ test, (c) fluorescence image of skin slice after the ‘press and release’ of the TMAP loaded with rhodamine B, and (d) the relationship between the percentage of successful insertions and microneedle number. Inserts: microscopic images of rabbit skin treated with TMAP at (d1) the 1st cycle, and (d2) 12th cycle; SEM images of (d3) microneedles and (d4) its bent microneedle after the 12th ‘press and release’ cycle.

### 2.8. In vivo transdermal insulin delivery for diabetic rats

All rats were fasted for 12 h but allowed to drink freely before the injection of STZ. The rat hair on the back region was shaved, as shown in Figure S3(a). Rats were intraperitoneally injected with a dose of 55 mg/kg STZ in citric acid buffer (pH 4.3, 10 mg/mL) to induce type-1 diabetes. The blood glucose levels (BGLs) of diabetic rats were monitored for 3 d. The rats whose BGL exceeded 300 mg/dL and maintained stable hyperglycemia were used for subsequent drug administrations (Yu et al., 2017a).

*In vivo* insulin delivery was provided for diabetic rats. Two drug administration approaches of TMAP were proposed to treat diabetic rats. Administration approach I (TMAP-I): the ‘press and release’ paradigm was executed five times per 30 min and was continually performed on diabetic rats during the entire transdermal delivery process. Administration approach II (TMAP-II): the ‘press and release’ was executed five times per 30 min and was subsequently performed for one first hour to lower the BGLs of diabetic rats. A cycle ‘press and release’ was repeated on diabetic rats immediately once an increase of measured BGL occurred in the normoglycemic state. The rats were divided into 11 groups (*n* = 4 for each group): (1) healthy rats without treatment, (2) diabetic rats without treatment, (3) diabetic rats treated by subcutaneous injection of 5 IU insulin, (4) diabetic rats treated by 5 IU-insulin-loaded TTP, (5) diabetic rats poked by solid MA, and patched rats with 5 IU-insulin-loaded TTP following removal of solid MA, (6) diabetic rats treated by 5 IU-insulin-loaded TMAP-I, (7) diabetic rats treated by 10 IU-insulin-loaded TMAP-I, (8) diabetic rats treated by 20 IU-insulin-loaded TMAP-I, (9) diabetic rats treated by 5 IU-insulin-loaded TMAP-II, (10) diabetic rats treated by 10 IU-insulin-loaded TMAP-II, and (11) diabetic rats treated by 20 IU-insulin-loaded TMAP-II. TMAP was taped on the back region, as shown in Figure S3(b). Rat blood was collected from the tail vein every hour during drug administration. The glucose concentrations were measured with a blood glucose meter (Sinocare Inc., Changsha, China).

### 2.9. Numerical simulation of transdermal drug delivery

Numerical simulation models were built by COMSOL Multiphysics to explain the transdermal drug delivery mechanism of TMAP. The transdermal insulin delivery model of TMAP and its mesh model of viable skin are shown in Figure S4. The specific parameters of the simulations are listed in Table S2. All simulation models were built based on the physical size of MA and drug reservoirs. The radius shrinkage rate of the microchannel in the skin was fitted with Equation (S1) based on the TEWL test results. The drug permeability curves of the microchannels administered with various approaches are shown in Figure S5. Detailed descriptions of simulations are presented in the Supporting Information section. The diffusion of insulin and its concentration distribution in the skin during the delivery process was calculated based on Fick's laws of diffusion. The diffusion flux and total diffusion amount of insulin into the skin were calculated accordingly.

**Figure 4.**
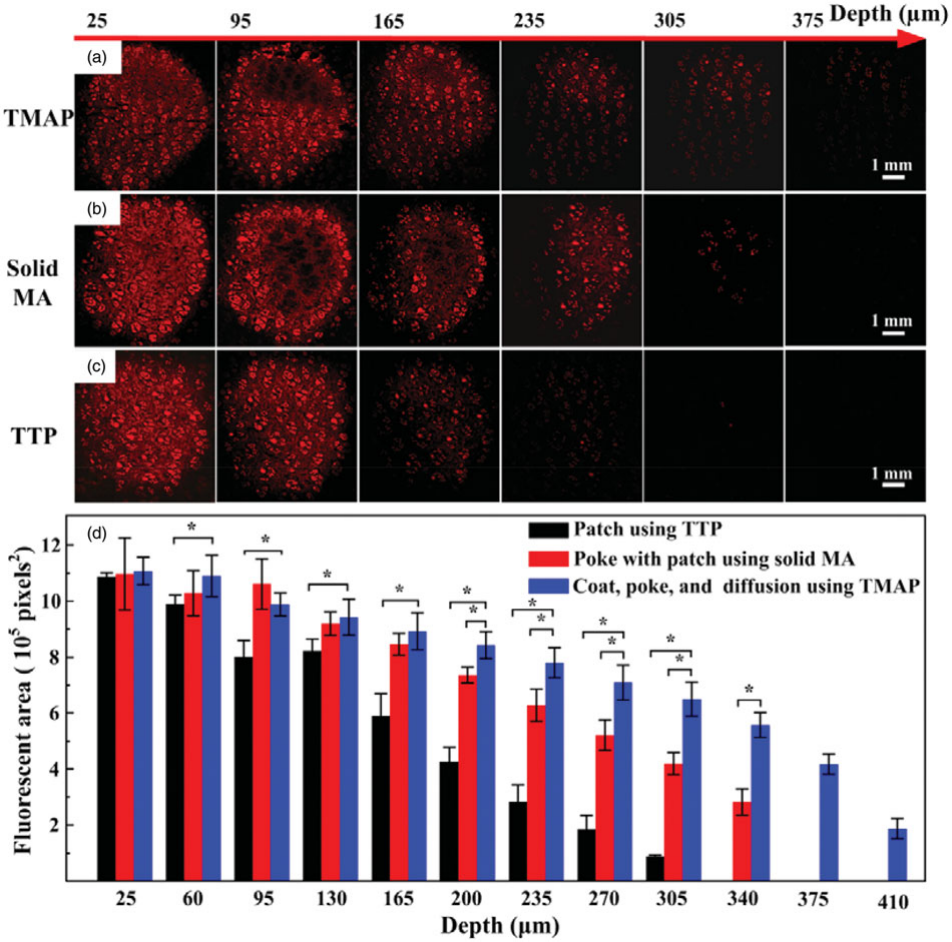
(a) *In vitro* transdermal delivery of rhodamine B using TTP, solid MA, and TMAP. (b) The calculated fluorescence area of diffused rhodamine B at various depths after application of TMAP in comparison with TTP and solid MA.

**Figure 5.**
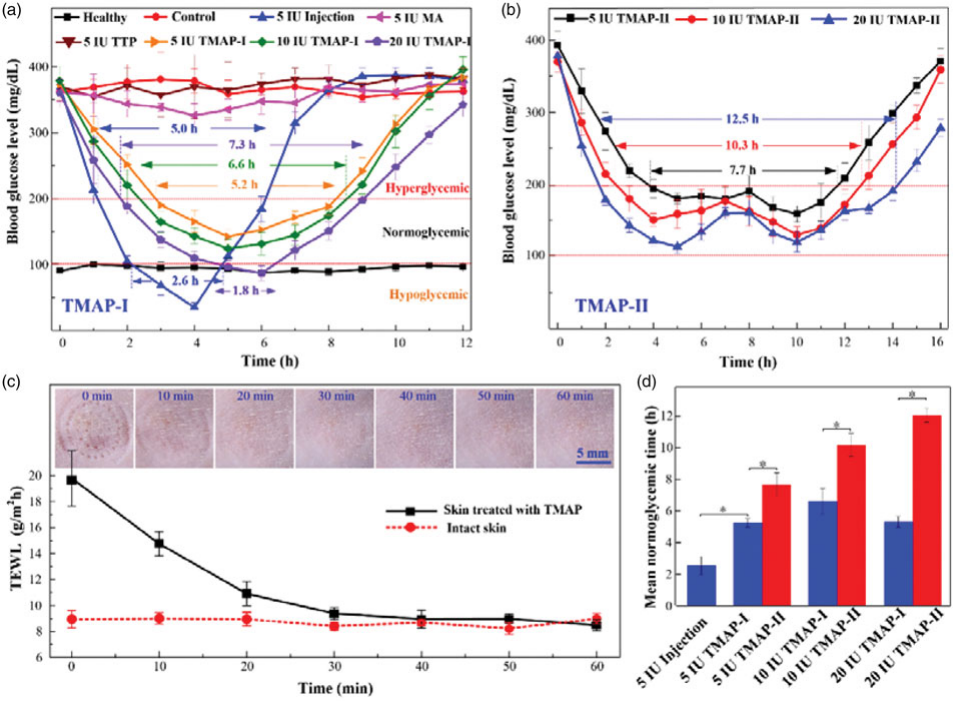
*In vivo* studies of insulin-loaded TMAP for type-1 diabetic rats treatment: (a) BGLs of diabetic rats administrated with TMAP-I, TTP, solid MA, and subcutaneous injection, (b) BGLs of diabetic rats administrated with TMAP-II, (c) mean TEWL values and recovery process of punctured skin after removal of TMAP, and (d) histogram of mean normoglycemic time as a function of various delivery approaches.

### 2.10. Statistical analysis

All experiments conducted in this study were repeated using at least four samples. The arithmetic mean and standard error of the mean were calculated from all data points, and were plotted on each graph. Student’s t-tests were conducted when the two groups were compared. *p* Values <.05 were considered statistically significant.

## 3. Results and discussion

### 3.1. Transdermal drug delivery mechanism

TMAP is developed for transdermal delivery of liquid drugs. Its expanded view is shown in Figure 1(a). It mainly consists of a medical tape, an anti-seepage gasket, a medical sponge, and a solid MA. The gasket, sponge, and MA, are concentrically bonded on the medical tape. The waterproof medical tape is flexible and can patch the TMAP closely on the skin. It can also minimize drug loss and impede the flux of toxins into the skin. The anti-seepage gasket is used to avoid the liquid drug from leakage during the compression of TMAP. The medical sponge, used as a drug reservoir, can load a substantial amount of liquid drug in its pores. Solid MA, used as a skin puncher, can poke through the SC layer and create microchannels for the passive diffusion of liquid drug formulation into the systemic circulation. In simple terms, TMAP is a typical TTP integrated with a solid MA for transdermal delivery of liquid drugs.

The transdermal drug delivery strategy of TMAP is shown in Figure 1(b–c). A liquid-drug-loaded TMAP is taped on the skin with a medical tape. TMAP is compressible because both the sponge and the gasket are elastic. Liquid drug can be delivered via the finger touch-actuated actions of ‘press and release,’ as shown in Figure 1(b). The detailed drug administration process of TMAP is presented in Figure 1(c). It mainly includes three key steps: ‘coat, poke, and diffusion.’ The solid MA is totally soaked in the medical sponge and coated with the liquid drug formulation. Since the TMAP is pressed with a finger, solid MA penetrates through the sponge and the SC layer into the skin tissue, thus creating transient aqueous microchannels. The drug formulation coated on MA is deposited and dissolved in the skin. As the compression of the finger on TMAP is released, the solid MA will detach from the skin and retract the sponge owing to the elastic rebound energy of the sponge and gasket. Microchannels are directly exposed in the liquid drug formulation of the sponge. The liquid drug formulation will passively permeate in the skin through the microchannels. However, the microchannels will shrink gradually owing to the recovery of the skin, and the SC layer barrier will return to its normal status within a certain period after the solid MA removal. The transdermal delivery process stops once the microchannels get self-closed. The patients can repeat the aforementioned touch-actuated ‘press and release’ process on the TMAP to reopen the microchannels for drug delivery again. The diffusion velocity of the liquid drug is greatly determined by the number of microchannels created by the solid MA and the liquid drug concentration in the sponge. The diffusion flux of the liquid drug can also be actively controlled by switching the microchannels in the skin to self-close and reopen. TMAP is easily used by nonskilled users and/or self-administered.

### 3.2. Characterization

According to the above design, a TMAP was assembled, as shown in Figure 2(a). The main components, including medical tape, gasket, MA, and sponge, can be clearly observed. Their specific parameters are listed in Table S1 in the Supporting Information section. The materials used to manufacture each of the components have been extensively used in clinical practice and have been approved by the Food and Drug Administration. The components are simple and have low cost. The fabrication cost of TMAP is less than 0.14 US dollars (USD). Moreover, TMAP can be easily disposed. TMAP taped on the back of a human hand is shown in Figure 2(b). The waterproof medical tape can match closely the curved human skin, maintaining a stable interface between TMAP and skin, and minimizing loss of liquid drug formulation. A PMMA solid MA fabricated using the micromolding technique is shown in Figure 2(c–d). PMMA has increased mechanical resistance, unlimited disposability, and biocompatibility (Cimatti et al., 2017; Paz et al., 2017). The solid MA consists of 42 conical microneedles, which are concentrically and uniformly arranged on the substrate. The SEM image of a microneedle is presented in Figure 2(e). The average height, tip diameter, and base diameter of the microneedle, are approximately 600 μm, 10 μm, and 180 μm, respectively. The microneedle’s surface is smooth and the tips are sharp for easy skin penetration. The SEM image of the medical sponge is shown in Figure 2(f). The sponge is the drug reservoir of TMAP. It is porous and contains numerous micropores that ranged from several micrometers to hundreds of micrometers, as observed. Solid MA is covered by the sponge, which can effectively prevent scratching by the microneedles and eliminate cross-contamination.

### 3.3. Storage performance

Most microneedle-based transdermal patches are usually limited by the restricted dose of delivered drug due to the finite size of the microneedles, while TMAP can adequately address this issue since it can store a large amount of macromolecular drug formulation in the medical sponge. The sponge can swell with water, and can load the liquid drug formulation in its pores by capillary action. The drug storage capacity of TMAP is mainly determined by the drug concentration, and the porosity and volume of the sponge. The drug concentration can be easily adjusted. In this study, the porosity of the medical sponge loaded with insulin solution was approximately 95%, in accordance to the density method measurements (Li et al., 2017a). The volume of the sponge in our TMAP was 194 mm^3^. The maximum loading capacity of TMAP without spilling was approximately 180 μL in this experiment, which is close to 184 μL predicted by theory. Therefore, TMAP has an excellent drug loading capacity.

TMAP can be directly applied in transdermal drug delivery by administering liquid drug in the sponge. A titration method can effectively avoid the heating step in the fabrication process of some MA, which may cause the breakdown of the heat-sensitive drug and eradicate its pharmacological activity. The drug-loaded TAMP also can be prepared *a priori* and stored, which makes self-administration possible for the patients. Thus, the stability of liquid drug formulation was investigated in TMAP. The biological activity of stored insulin in TMAP was measured using an insulin ELISA kit (Jianglai industrial Ltd., Shanghai, China), as shown in Figure 3(a). The insulin gradually denaturized with time and temperature. Approximately 96%, 93%, and 90%, of insulin doses maintain their biological activities in TMAPs stored at 4 °C for 5 d, 15 d, and 25 d, respectively. However, most of the insulin that was stored at room temperature (approximately 25 °C) denaturized within 5 d. Therefore, insulin-loaded TMAP could maintain good bioactivity within 25 d if stored at a temperature of 4 °C.

### 3.4. Mechanical performance

Solid MA of TMAP must penetrate through the 15–20 μm thick SC layer and retract from the skin to create microchannels for passive permeation of liquid drug without breakage (Chen et al., 2018). Figure 3(b) shows the resistance force of TAMP during the ‘press and release’ test. The resistance force gradually increases with the loading displacement owing to the inherent elastic resistance of skin and TMAP. As the compression further proceeds, the microneedle tips poke on the skin, and generate extremely high stress concentrated on the contact points. As the increasing stress reaches the rupture limit of skin, microneedle tips penetrate into the skin, resulting in a sudden drop of the resistance force at point ‘P_1_.’ According to previous reports, this is the critical force needed to achieve microneedle penetration into the skin (Khanna et al., 2010; Cho et al., 2012). The penetration force of TMAP is 1.2 N and the energy required for this penetration is 5.95 mJ. The average compression force of an adult pressing the microneedles with his or her thumb is approximately 20 N (Larrañeta et al., 2014). Therefore, TMAP can easily penetrate into the skin using a finger compression.

As the compression force is gradually released, the resistance force decreases as a function of the unloading displacement. This unloading process is in a quasi-static state. Consider the TMAP as the analysis object. Ignoring the effect of medical tape, the measured resistance force of TMAP is approximately equal to the combined elastic recovery force of the skin and TMAP minus the friction force between the skin and microneedles. Once the microneedles are detached from the skin owing to the elastic rebound energy of the sponge, the friction force becomes zero, thereby resulting in an increase of the measured resistance force at point ‘P_2_.’ The resistance force at point ‘P_2_’ is approximately 1 N. Once the microneedles are detached from the skin, the microneedles are gradually retracted in the sponge. The resistance force further decreases with the elastic recovery of the TMAP and skin.

Figure 3(c) shows the fluorescence image of the skin slice after ‘press and release’ using a TMAP loaded with rhodamine B. The outermost layer of the skin is the SC layer and the underlying thin layer is the viable epidermis, which is seen atop of a thick layer of dermis. A punctured hole with a penetration depth of approximately 190 μm and a base width of approximately 80 μm can be clearly observed. This further demonstrates that the microneedles of TMAP were completely penetrated through the layers of SC and the epidermis into the dermis layer. The fluorescence of rhodamine B in the dermis layer was mostly distributed along the edge of the hole. This indicated that the liquid drug of TMAP could be permeated in the dermis layer. Above all, TMAP can penetrate through the SC layer and retract from the skin to create microchannels.

TMAP delivers liquid drug by repeated ‘press and release’ actions. Thus, it is important to assess the mechanical stability of TMAP (i.e. the ability to create multiple insertions into the skin). The mechanical stability of TMAP can be evaluated by the relationship between the intact insertions and the microneedle number (Li et al., 2017b). The red dots remained on the skin surface indicates the rhodamine B permeation into the skin through the microchannels created by solid MA, which means successful insertion. As shown in Figure 3(d), TMAP achieved almost 100% successful insertions in the first seven cycles (with five successive ‘press and release’ repetitions at one spot of the skin being considered as a cycle), thereby indicating the good initial mechanical property of the microneedles. Further increases in the insertion cycles on the rabbit skin resulted in a decrease of the percentage of successful insertions. The percentage of successful insertions decreased to approximately 95% at the 12th cycle. The microneedles of TMAP after the 12th ‘press and release’ cycle were observed, as shown in Figure 3(d3–d4). Several microneedles were slightly bent without breakage, which possibly resulted in the failure of the microneedles to insert into the skin. It also indicated that PMMA solid microneedles had good toughness, reducing the risk of breakage in the skin. Therefore, solid microneedles of TMAP exhibited good mechanical stability for repeated ‘press and release’.

### 3.5. *In vitro* transdermal drug delivery performance

*In vitro* transdermal delivery of rhodamine B using TMAP via ‘press and release’ was investigated to evaluate its feasibility of efficient delivery for drugs comprising small molecules in comparison with TTP and solid MA, as shown in Figure 4. The fluorescence area and intensity of diffused rhodamine B gradually decreased with depth after 30 min of diffusion. The maximum depths of diffusion of rhodamine B using TMAP, solid MA, and TTP, were approximately 410 μm, 340 μm, and 305 μm, respectively. Rhodamine B of TTP could slowly diffuse through the SC layer owing to its small molecular weight (approximately 479 Da). Solid MA could assist rhodamine B across the SC layer by creating microchannels in the skin, enhancing permeation at greater depths. Compared with solid MA, TMAP exhibited a faster transdermal diffusion rate, resulting in a deeper diffusion depth. The possible reason is that the ‘press and release’ paradigm further enhanced the delivery performance by repeatedly opening microchannels for permeation and by carrying coated drug for dissolution in the skin. Therefore, TMAP exhibited good potential to assist pharmaceutical transportation and enhance drug diffusion efficiency across the SC layer.

### 3.6. In vivo transdermal insulin delivery in diabetic rats

Diabetes is one of the leading lethal diseases and insulin is the most effective medicine to control BGL in type 1 diabetic patients. Transdermal delivery of insulin has been regarded as an attractive alternative owing to its easy self-administration and good patient compliance (Xie et al., 2015). To investigate the *in vivo* transdermal delivery performance of TMAP, insulin-loaded TMAP was operated on the STZ-induced type-1 diabetic rats, as shown in Figure 5. Blood glucose concentrations of SD rats in the range of 100 mg/dL–200 mg/dL were defined to be in the normoglycemic state (Yu et al., 2017a; Yu et al., 2017c). The effective time periods during which diabetic rats were in normoglycemic states were defined as the normoglycemic times.

Figure 5(c) presents the mean TEWL values and recovery ability of punctured skin following the removal of TMAP on SD rats. TEWL measurements, used extensively to evaluate the skin barrier function, showed that the skin barrier returned to its normal state within 30 min of TMAP removal, regardless of the treatment protocol. The micropores punctured by TMAP could be clearly observed on the rat skin after TMAP removal, while they gradually disappeared within 30 min. Above all, the punctured skin can self-heal and micropores close within 30 min, resisting the transdermal delivery of liquid drug. Therefore, we chose to repeat the ‘press and release’ experiment every 30 min to reopen the microchannels for liquid drug permeation.

Figure 5(a) presents the BGLs of rats administrated with insulin using different transdermal delivery approaches. The BGLs of the health and blank groups were maintained stable at 94.6 ± 8.8 mg/dL and 378.6 ± 18.0 mg/dL, respectively. The BGLs of diabetic SD rats treated by using 5 IU-insulin-loaded TTP were not lower. Insulin cannot passively pass through the intact SC layer owing to its high molecular weight of 5.8 kDa (Xie et al., 2015). The BGLs of diabetic rats treated using a solid MA and a ‘poke with patch’ approach decreased slightly. The insulin dose that diffused in the skin was limited owing to the self-closure of the microchannels. Comparatively, both insulin-loaded TMAP and subcutaneous injection exhibited strong hypoglycemic effects. The BGLs of diabetic rats injected with 5 IU-insulin decreased rapidly to the minimum value of 35.1 ± 6.97 mg/dL within 4 h, and quickly rebounded to the initial level after 5 h. The BGLs of the subcutaneous injection group were in a hypoglycemic state for 2.6 h, thereby inducing a risk of hypoglycemia (Yasuo Ohkubo, 1995). The normoglycemic time treated with subcutaneous injection was approximately 2.4 h. The BGLs of diabetic rats treated with 5 IU-insulin-loaded TMAP-I decreased slowly to attain its lowest value (152.2 mg/dL), was then maintained at the normoglycemic state for 5.2 h, and then gradually increased to its initial level. The normoglycemic time period spanned 5.2 h, which was more than twice the amount administrated by subcutaneous injection. Above all, 5 IU-insulin-loaded TMAP-I exhibited the best hypoglycemic effect on the diabetic rats.

The transdermal insulin delivery performance can be improved by increasing the insulin dosage loaded in TMAP-I, as shown in Figure 5(a). The BGLs of diabetic rats decreased quickly as a function of insulin dosage of TMAP-I. This indicated that more insulin was diffused in the body at the initial stage with higher concentrations of insulin. The normoglycemic times treated with 5 IU, 10 IU, and 20 IU insulin-loaded TMAP-I were 5.2 h, 6.6 h, and 5.5 h, respectively. The application of 20 IU-insulin-loaded TMAP-I induced the rats in a hypoglycemic state for 1.8 h, and may lead to increased morbidity. Therefore, 10 IU-insulin-loaded TMAP administrated with approach I is the optimal choice for the treatment of diabetic rats.

A ‘closed-loop’ administration approach of TMAP was proposed for on-demand dosage control to further improve the hypoglycemic effect. The detailed ‘closed-loop’ administration process of 5 IU, 10 IU, and 20 IU insulin-loaded TMAP-II are presented in Figure S6 of the Supporting Information section. As the blood glucose meter detected an increase of BGL in the normoglycemic state, ‘press and release’ actions were performed to reopen the microchannels through the SC barrier layer for the delivery of the insulin solution. The BGL usually declined again. Therefore, the BGLs were well controlled and fluctuated in a reasonable range. This is the ‘closed-loop’ administration approach II of TMAP. The BGLs that use the ‘closed-loop’ administration approach II are presented in Figure 5(b). The normoglycemic times treated with 5 IU, 10 IU, and 20 IU insulin-loaded TMAP-II were 7.7 h, 10.3 h, and 12.5 h, respectively. The normoglycemic time was significantly prolonged, and the number of ‘press and release’ actions on TMAP were obviously decreased. Compared with subcutaneous injection, the insulin in TMAP-II was passively and gradually diffused through microchannels in the skin through rapid injection. Therefore, the risk of induction of diabetic rats in a hypoglycemic state was effectively avoided by the application of TMAP with administration approach II.

Compared with the subcutaneous injection and TMAP-I, TMAP-II exhibited the best hypoglycemic effect and the mean normoglycemic time was obviously enhanced, as shown in Figure 5(d). The mean normoglycemic time treated with 5 IU injections, 5 IU-insulin-loaded TMAP-I, and 5 IU-insulin-loaded TMAP-II, were 2.55 h, 5.25 h, and 7.68 h, respectively. The mean normoglycemic time treated with 20 IU-insulin-loaded TMAP-II was approximately 11.63 h. This indicated that just two TMAPs could maintain the diabetic rats in the normoglycemic state for a day. To the best of our knowledge, this is the longest normoglycemic time period achieved with treatments using the insulin-loaded microneedle-based transdermal patches, as reported for type 1 diabetic rats (Chen et al., 2009; Zhou et al., 2010; Liu et al., 2012; Ling and Chen, 2013; Li et al., 2017b; Yu et al., 2017a; Yu et al., 2017b; Yu et al., 2017c). Therefore, 20 IU-insulin-loaded TMAPs administrated with approach II is an attractive candidate to treat type 1 diabetic SD rats.

**Figure 6.**
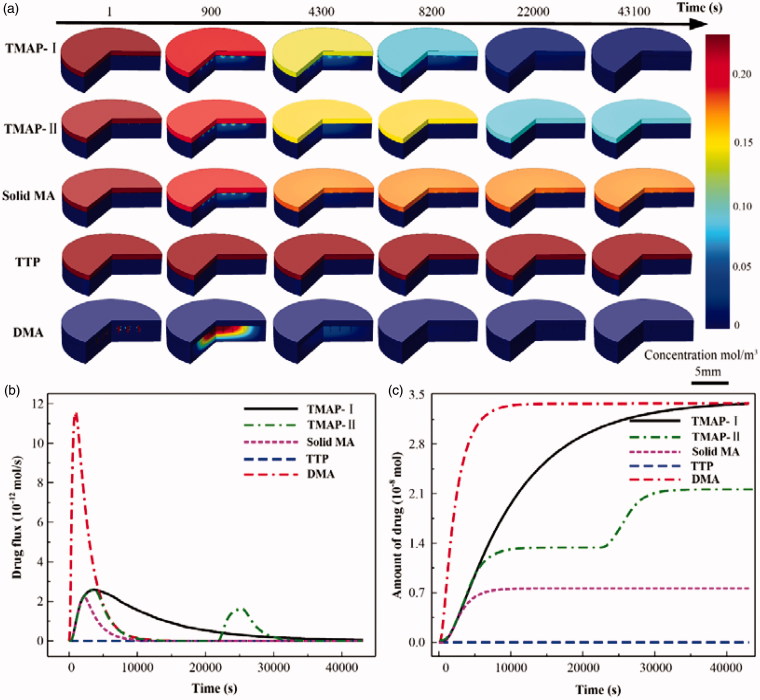
The simulation results of transdermal insulin delivery administrated with 5 IU-insulin-loaded TMAP-I, TMAPII, solid MA, TTP, and DMA, using COMSOL: (a) the concentration distribution diffused in the skin during the transdermal insulin delivery process, (b) the diffusion flux of insulin into the skin, and (c) the total diffusion amount of insulin into the skin.

The transdermal insulin diffusion process administrated with 5 IU-insulin-loaded TMAP-I, TMAP-II, solid MA, TTP, and DMA, were numerically simulated with COMSOL, as shown in Figure 6(a). Accordingly, the flux and total amount of insulin permeated into the skin were calculated, as shown in Figure 6(b–c). The diffusion flux and amount of insulin administrated with TTP is almost zero. The transdermal delivery of macromolecular drugs using TTP is mainly resisted by the SC layer. The drug delivery approach of the solid MA is ‘poke with patch’. Permeation through microchannels occurs and stops within periods of 30 min owing to the self-closure of microchannels. The typical delivery approach of DMA is ‘dissolve and release.’ A pulse diffusion flux of insulin can be observed whereby the utilization of the insulin is maximized. Microneedles of DMA are rapidly dissolved by the interstitial fluid, releasing insulin to lower the BGL. The reported normoglycemic time of 5 IU-insulin-loaded DMA treated on type 1 diabetic SD rats was approximately 3.5 h. Approximately 1.5-fold and 2.2-fold increases were documented in the normoglycemic time administered with 5 IU-insulin-loaded TMAP-I and TMAP-II, respectively. Compared with DMA, the insulin of TAMP-I diffuses into the skin at a relatively slow rate to lower BGL via the ongoing self-closure and reopening of microchannels via the ‘press and release’ actions. The diffusion flux of insulin administered with TMAP-II can be tuned in a closed-loop control mode by switching the microchannels to self-close and reopen based on the feedback of BGLs. Therefore, therapeutic effects can be enhanced and on-demand dosage control can be achieved by this ‘closed-loop’ administration approach. Furthermore, in the future, we will develop a TMAP integrated with microneedle-based biosensor for glucose measurement to treat diabetes in a ‘closed-loop’ control mode.

## 4. Conclusions

This study developed a novel microneedle-based transdermal patch referred to as TMAP for long-term delivery of liquid macromolecular drugs. TMAP is a combination of TTP and solid MA. TMAP is simple and suitable for mass fabrication at a low-cost of less than USD 0.14. TMAP has a drug reservoir, which can be easily loaded with a high dose of liquid drugs, particularly with thermosensitive macromolecular drugs. The insulin stored in TMAP at a temperature of 4 °C can maintain good bioactivity within a period of 25 d. The delivery strategy of TMAP is in accordance to the principle of ‘coat, poke, and diffusion.’ TMAP can easily penetrate through the SC layer and retract from the skin to create microchannels using touch-actuated ‘press and release’ actions. TMAP exhibits good mechanical stability that could achieve approximately 95% successful insertions without microneedle breakage after 12 consecutive ‘press and release’ cycles. TMAP loaded with rhodamine B exhibited the fastest transdermal diffusion rate and deepest diffusion depth in comparison to the solid MA and TTP. Insulin-loaded TMAP with two administration approaches were developed to treat type-1 diabetic SD rats. Insulin-loaded TMAP-I exhibited the best hypoglycemic effect on the diabetic rats in comparison with TTP, solid MA, subcutaneous injection, and DMA. TMAP-II can provide insulin delivery in on-demand dosage control. The diffusion flux of insulin can be adjusted in a closed-loop control mode by switching the reopening and self-closure of the permeable microchannels based on feedback of BGLs. The mean normoglycemic time treated with 20 IU-insulin-loaded TMAP-II was approximately 11.63 h, and is the longest normoglycemic time reported in microneedle-based transdermal patches reported to date. Therefore, TMAP is expected to have broad application prospects in the pharmaceutical field.

## Supplementary Material

Supplemental Material
