# Supplementary material for: Touch-actuated microneedle array patch for closed-loop transdermal drug delivery
Source: Drug Deliv. 2018 Sep 5;25(1):1728–39. doi: 10.1080/10717544.2018.1507060 (PMC6127806; doi:10.1080/10717544.2018.1507060)
Supplement: Supplemental Material [file IDRD_A_1507060_SM6697.docx]

Supporting Information for

**Touch-actuated Microneedle Array Patch for Closed-loop Transdermal Drug Delivery**

Jingbo Yang ^1^, Zhipeng Chen ^1^, Rui Ye ^1^, Jiyu Li ^1, 2^, Yinyan Lin ^1^, Jie Gao ^1^, Lei Ren ^1^, Bin Liu ^1^, Lelun Jiang ^1,^ *

1. Guangdong Provincial Key Laboratory of Sensor Technology and Biomedical Instrument, School of Biomedical Engineering, Sun Yat-Sen University, Guangzhou, PR China;
2. Department of Mechanical and Biomedical Engineering, City University of Hong Kong, Hong Kong,
PR China;

# The parameters of TMAP components

TMAP is mainly composed of medical tape, anti-seepage gasket, medical sponge and solid MA. The specific parameters of four components are listed in Table S1. The cost of TMAP is less than 0.14 dollar.

Table S1 The specific parameters of TMAP components

| **Components** | **Materials** | **Size** | **Price ($)** |
| --- | --- | --- | --- |
| Medical tape | PU film with adhesive | Diameter: Φ 40 mm;  Thickness: 0.05 mm. | < 0.04 |
| Anti-seepage gasket | EVA foam | Inner circle diameter: Φ15mm;  Outer circle diameter: Φ22 mm;  Thickness: 1.1 mm. |  |
| Medical sponge | Polyvinyl alcohol | Porosity ˃ 95 %;  Diameter: 15 mm;  Thickness: 1.1 mm. | < 0.02 |
| Solid MA | PMMA | Microneedle number: 42;  Substrate diameter: 12 mm;  Microneedle height: 0.6 mm;  Microneedle base diameter: 0.18 mm. | < 0.08 |

# Fabrication of PMMA solid MA by micromolding technique

Methyl methacrylate (MMA) and benzoyl peroxide (BPO) were purchased from Zhiyuan Chemical Reagent Co., Ltd, China. Polydimethylsiloxane (PDMS, Sylgard 184) was bought from Dow Corning, England. Micromolding technique was proposed to fabricate solid MA. Micromolding is suitable for mass production. The detailed fabrication process is presented in Fig. S1.

(1) Fabrication of PDMS female mold: a commercial MA stamp (DRS50, DRS Dermaroller Company, Germany) was purchased as a master template. This MA template consisted of 42 conical microneedles with a length of approximately 600 μm and a base diameter of 180 μm. Inverse replicas of master template was fabricated from PDMS at a 10:1 ratio of pre-polymer to curing agent. The detailed fabrication process of PDMS female mold was performed, according to the previous report (Bediz *et al.*, 2014).

(2) Fabrication of PMMA solid MA: MMA was uniformly mixed with BPO at a weight ratio of 71.4: 1 to prepare the pre-polymerized PMMA at a temperature of 80 °C for 40 min. The pre-polymerized PMMA was cast onto the PDMS mold under a vacuum of 2000 Pa for a night. The PMMA was solidified in the PDMS mold. The PMMA solid MA was peeled off from PDMS mold and finally fabricated.


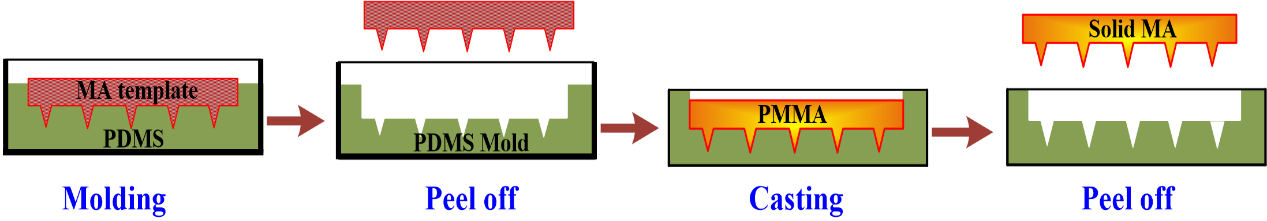


Figure S1. Fabrication process of PMMA solid MA by micromolding technique

# Mechanical loading setup for poke and release test

A custom-made mechanical loading setup was developed for the investigation of “press and release” performance of TMAP, as shown in Fig. S2. A force sensor (Nano 17 Titanium, ATI Industrial Automation, USA) is assembled on a DC linear motor (E-861, PI, German). The force range and resolution of force sensor are 14.1 N and 2.93 mN, respectively. The loading force and displacement during the “press and release” test can be collected by the force sensor and linear motor, respectively.


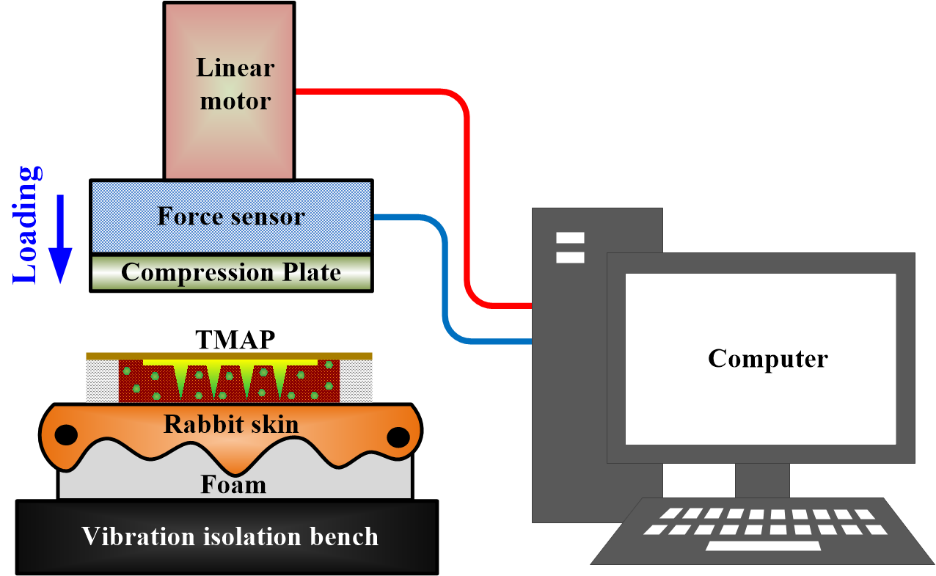


Figure S2. Schematic illustration of mechanical loading setup for the “press and release” test of TMAP

# Diabetic SD rats

As shown in Fig. S3, the SD diabetic rats with a weight of 210 ± 30 g caused by STZ were chosen as animal models. After the rats were shaved off their back, the insulin-loaded TMAP was patched onto the naked area for transdermal drug delivery and TEWL test.


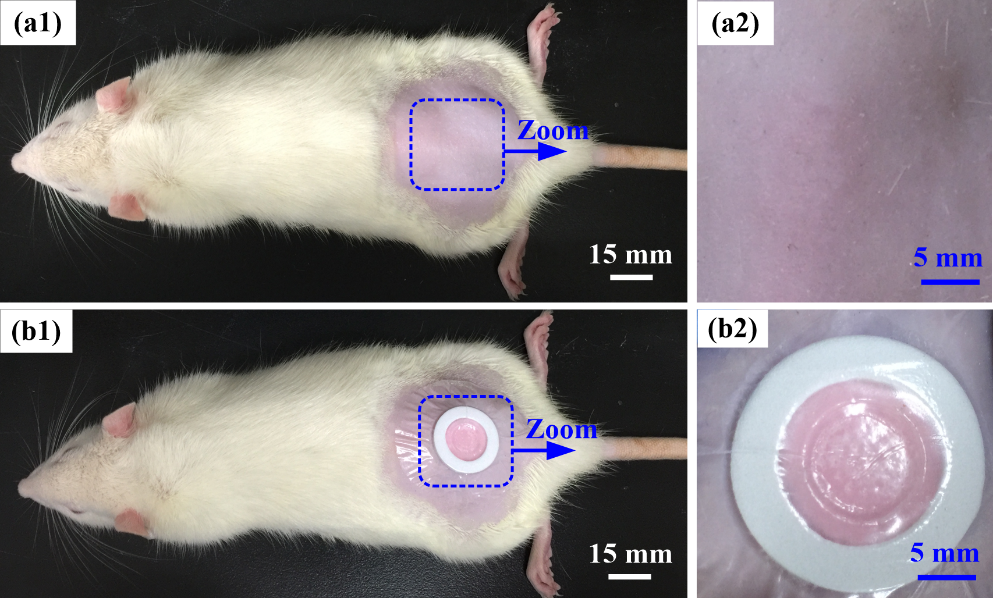


Figure S3. Images of experimental SD rats and placement position of insulin-loaded TMAP

# Numerical simulation of transdermal drug delivery

The Fick's laws of diffusion were employed to numerically calculate the transdermal diffusion of insulin into the skin administrated with TMAP-I, TMAP-II, TTP, solid MA and DMA. A 1/6 physical model of TMAP transdermal insulin delivery and the mesh model of viable skin by COMSOL are shown in Fig. S4. All simulation models of various administration approaches were built based on the physical size of MA and drug reservoir. The specific parameters of simulations were listed in Table S2. The stratum corneum layer is impermeable for macromolecular drugs. Thus, the permeability of stratum corneum layer was set as 0. The diffusion coefficient in viable skin was set to 1.2×10^-10^ m^2^/s (Leeladurga *et al.*, 2016, Uppuluri *et al.*, 2017). The liquid drug was continuously transported through the bottom of skin to the systemic circulation. The drug diffusion concentration at the bottom of skin was set as 0. The microchannels created by MA were gradually self-healing, resulting in a self-closure. The radius shrinkage rate of microchannel *r* with diffusion time *t* can be fitted by Eq. (S1) according to the TEWL [experiment](file:///F:\360Downloads\Youdao\Dict\7.2.0.0703\resultui\dict\?keyword=experiment) result.

*r* =1- *t* /1800 (S1)

Once the “press and release” is performed, the microchannels are opened, and the drug diffusion occurs. The drug permeability curves via microchannels are shown in Fig. S5.


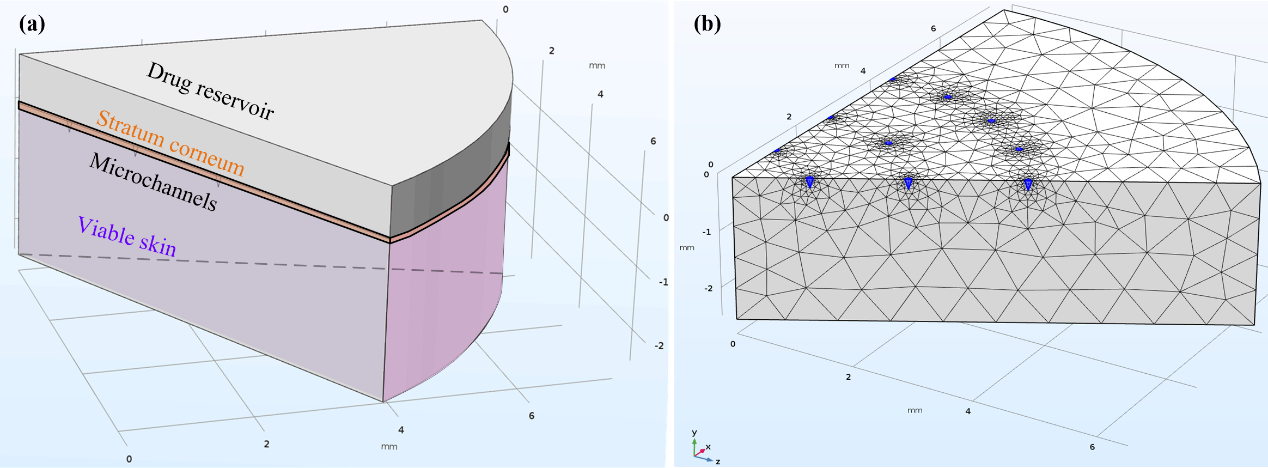


Figure S4. (a) 1/6 simulation model of TMAP transdermal insulin delivery, and (b) the mesh model of viable skin.

Table S2 The specific parameters for numerical simulations

| **Parameters** | **TMAP** | **Solid MA** | **TTP** | **DMA** |
| --- | --- | --- | --- | --- |
| Sponge radius (mm) | 7.5 | 7.5 | 7.5 | None |
| Sponge thickness (mm) | 1.1 | 1.1 | 1.1 | None |
| Skin radius (mm) | 7.5 | 7.5 | 7.5 | 7.5 |
| Skin thickness (mm) | 2.5 | 2.5 | 2.5 | 2.5 |
| Microneedles number | 42 | 42 | None | 42 |
| Microneedle base radius (µm) | 90 | 90 | None | 90 |
| Microneedle height (µm) | 600 | 600 | None | 600 |
| Drug [concentration](file:///F:\360Downloads\Youdao\Dict\7.2.0.0703\resultui\dict\?keyword=concentration) (mol/m^3^) | 0.234 (5IU) | 0.234 (5IU) | 0.234 (5IU) | 65.168 (5IU) |
| Diffusion coefficient (m^2^/s) | 1.2×10^-10^ | 1.2×10^-10^ | 1.2×10^-10^ | 1.2×10^-10^ |
| [Duration](javascript:;) (s) | 4.32×10^4^ | 4.32×10^4^ | 4.32×10^4^ | 4.32×10^4^ |


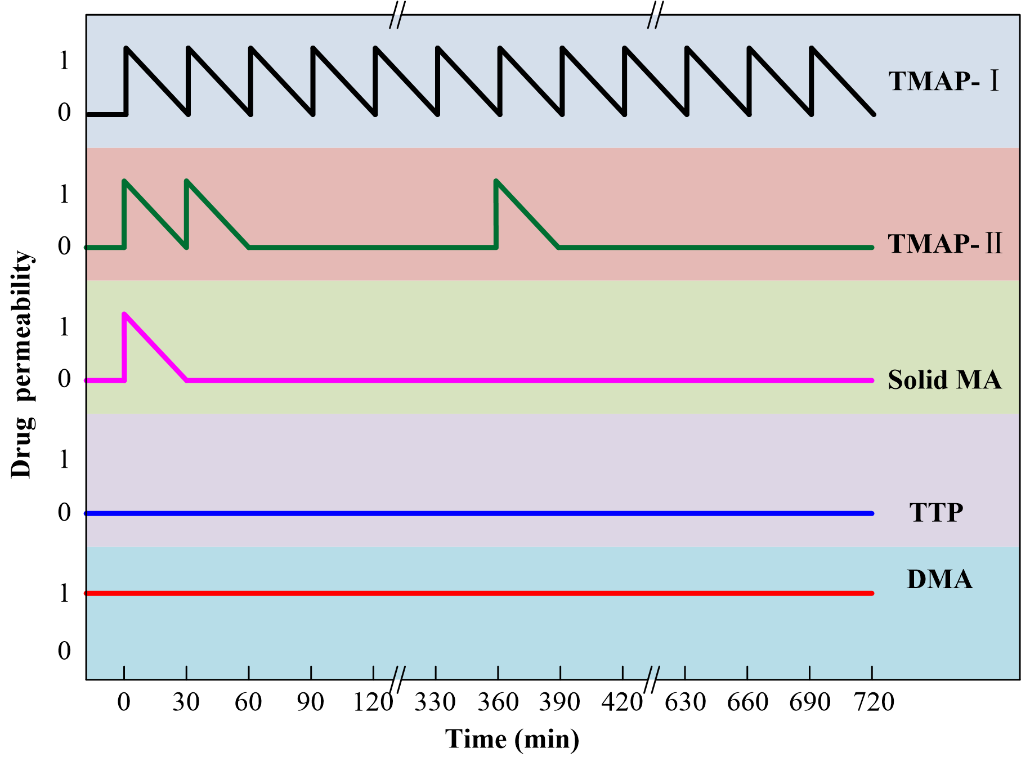


Fig. S5 The curves of drug permeability via microchannels administrated with different delivery approaches.

# “Closed-loop” administration approach II for diabetic rats

Fig. S6 (a-c) presents the BGLs of four diabetic rats administrated with (a) 5 IU-insulin-loaded TMAP, (b) 10 IU-insulin-loaded TMAP, and (c) 20 IU-insulin-loaded TMAP, respectively. Once the blood glucose meter detected an increase of BGL in normoglycemic state, “press and release” was performed on TMAP to lower the BGL again, adjusting the BGLs in the range of normoglycemic state. This is a BGL responsive “closed-loop” administration approach Ⅱ of TMAP.


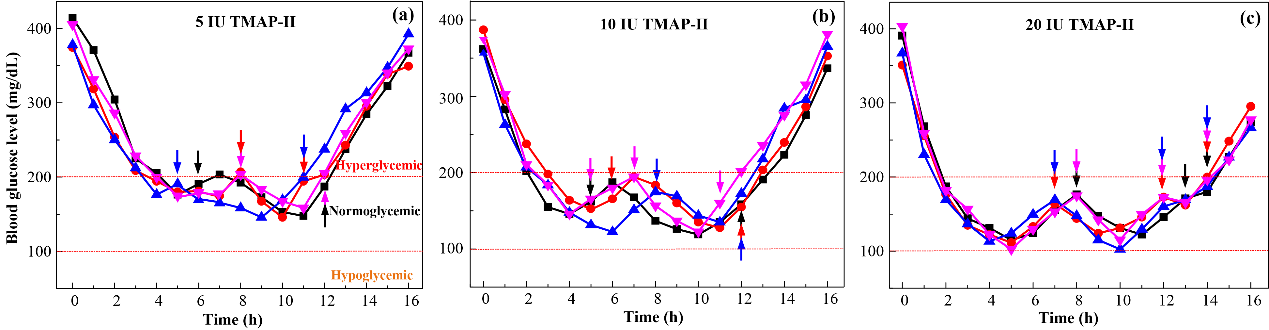


Fig. S6 the BGLs of four diabetic rats administrated with (a) 5 IU-insulin-loaded TMAP-II, (b) 10 IU-insulin-loaded TMAP-II, and (c) 20 IU-insulin-loaded TMAP-II. The arrow means a cycle of “press and release”.

# References

Bediz, B., Korkmaz, E., Khilwani, R., Donahue, C., Erdos, G., Falo, L.D. & Ozdoganlar, O.B., 2014. Dissolvable Microneedle Arrays for Intradermal Delivery of Biologics: Fabrication and Application. *Pharmaceutical Research,* 31**,** 117-135.

Leeladurga, V., Teja, U.C., Sultana, S.K.A., Sudeep, K., Anusha, V.S.S., Han, T., Nalluri, B.N. & Das, D.B., 2016. Application of Microneedle Arrays for Enhancement of Transdermal Permeation of Insulin: In Vitro Experiments, Scaling Analyses and Numerical Simulations. *Aaps Pharmscitech,* 17**,** 915-922.

Uppuluri, C.T., Devineni, J., Han, T., Nayak, A., Nair, K.J., Whiteside, B.R., Das, D.B. & Nalluri, B.N., 2017. Microneedle-assisted transdermal delivery of Zolmitriptan: effect of microneedle geometry, in vitro permeation experiments, scaling analyses and numerical simulations. *Drug Development and Industrial Pharmacy,* 43**,** 1292-1303.
